# Robotic Applications in Orthodontics: Changing the Face of Contemporary Clinical Care

**DOI:** 10.1155/2021/9954615

**Published:** 2021-06-16

**Authors:** Samar Adel, Abbas Zaher, Nadia El Harouni, Adith Venugopal, Pratik Premjani, Nikhilesh Vaid

**Affiliations:** ^1^Faculty of Dentistry, Alexandria University, Alexandria, Egypt; ^2^Department of Orthodontics, Saveetha Dental College and Hospitals, Saveetha Institute of Medical and Technical Sciences, Saveetha University, Chennai, India; ^3^Department of Orthodontics, University of Puthisastra, Phnom Penh, Cambodia; ^4^Swami Vivekananda Subharati University, Meerut, India; ^5^European University College, DHCC, Dubai, UAE

## Abstract

The last decade (2010-2021) has witnessed the evolution of robotic applications in orthodontics. This review scopes and analyzes published orthodontic literature in eight different domains: (1) robotic dental assistants; (2) robotics in diagnosis and simulation of orthodontic problems; (3) robotics in orthodontic patient education, teaching, and training; (4) wire bending and customized appliance robotics; (5) nanorobots/microrobots for acceleration of tooth movement and for remote monitoring; (6) robotics in maxillofacial surgeries and implant placement; (7) automated aligner production robotics; and (8) TMD rehabilitative robotics. A total of 1,150 records were searched, of which 124 potentially relevant articles were retrieved in full. 87 studies met the selection criteria following screening and were included in the scoping review. The review found that studies pertaining to arch wire bending and customized appliance robots, simulative robots for diagnosis, and surgical robots have been important areas of research in the last decade (32%, 22%, and 16%). Rehabilitative robots and nanorobots are quite promising and have been considerably reported in the orthodontic literature (13%, 9%). On the other hand, assistive robots, automated aligner production robots, and patient robots need more scientific data to be gathered in the future (1%, 1%, and 6%). Technological readiness of different robotic applications in orthodontics was further assessed. The presented eight domains of robotic technologies were assigned to an estimated technological readiness level according to the information given in the publications. Wire bending robots, TMD robots, nanorobots, and aligner production robots have reached the highest levels of technological readiness: 9; diagnostic robots and patient robots reached level 7, whereas surgical robots and assistive robots reached lower levels of readiness: 4 and 3, respectively.

## 1. Introduction

During the last decade, with the evolution of advanced manufacturing technologies, researching and designing together with the expanding popularity of three-dimensional (3D) imaging modalities, the implementation of robots has made a remarkable advance that has crept into every technological aspect including industrial fields, manufacturing processes, military purposes, medical fields, and research in which orthodontics is no exception. The inherent advantages of robots are their high accuracy and precision, high work efficiency, and stability [1].

Robotics is an interdisciplinary field that integrates computer science and engineering. Robotics is defined as the “intelligent connection between perception and action” [2]. The term robotics was introduced by writer Isaac Asimov in his science fiction book, *I, Robot*, published in 1950 [3]. According to the Robot Institute of America, a robot is defined as “a reprogrammable, multifunctional manipulator designed to move materials, parts, tools, or specialized devices through various programmed motions for the performance of a variety of tasks” [4].

There are many different types of robots that are used in divergent environments and for multiple uses. Although being very diverse in application and form, they all share three basic similarities when it comes to their construction: (1) all robots have some kind of mechanical construction to achieve a particular task, (2) robots have electrical components that power and control the machinery, and (3) robots contain some level of computer programming code which decides when or how to do something [5].

The discipline of orthodontics, since its very inception, has strived to improve the efficacy and efficiency of any kind of treatment delivered to patients. There are numerous enabler smart technologies in robotics that may contribute to the evolution of novel practices in orthodontics. This is especially true in today's new norms of social distancing and remote monitoring after the COVID-19 breakthrough. The pandemic has immensely emphasized the value of robotic implementation in orthodontics, since the risk of orthodontists being infected from COVID-19 secondary to aerosol exposure from an asymptomatic yet positive patient is quite high [6].

Current technological infrastructure in dental offices can be augmented by making use of a smart robot. By attempting to reduce the load, mentally and physically on human assistants, we can target the human factor. Robotic assistants can work tirelessly and can repeat a programmed workflow allowing humans to fulfill other tasks that robots are not able to do, such as direct social interaction with patients, diagnosis and treatment planning, or other work with high cognitive requirements [7].

We are living in an era of complete digital transformation of orthodontic records and 3D simulation of a patient's own problems to reach a correct diagnosis. In the context of robotics, this entails the use of robots for more accurate X-ray imaging and positioning [8, 9]; robotic automated 3D cephalometric annotation [10]; bionic robots for simulation of the stomatognathic system including masticatory and swallowing robots [11, 12], tongue robots [13], mandibular [14] and condylar movement simulation robots [15, 16], and dental articulation robots [17]; and robotic remote control of mandibular advancement appliances in obstructive sleep apnea (OSA) patients in order to efficiently reach the effective target protrusive position [18, 19].

With the entire clinical orthodontic field witnessing a conceptual and technological revolution, education and training are not an exception, where robotics have reinforced the casual educational teaching and training [20]. This goes back to 1969 when the idea of a dental training robot was first described [21]. In addition, there have been a number of attempts at developing masticatory robots for the purpose of providing dental patient training since the early 1990s [22]. Its heuristic value lies in the fact that it is able to perform actual mastication thereby enabling one to understand different scenarios, explore different ideas, develop novel hypotheses, and gain insight into the consequences of variations in masticatory function between and within individuals [23].

The last decade, in particular, has marked magnificent growth in the field of robotic wire bending and robotic customization of CAD/CAM appliances, increasing the effectiveness and efficiency of arch wire bending and treatment. The first developed wire bending robot was the Sure Smile robot by Butscher et al. in 2004 [24]. This was followed by other developed robots used by different labial and lingual customized CAD/CAM appliances like the Sure Smile [25–27], Incognito [28], LAMDA [29], Insignia [30–33], and BRIUS appliances [34].

*Nanotechnology* has printed profound breakthroughs in orthodontics during the last decade achieving efficient and effective treatment results [35]. This comprises nanomaterials, nanobiotechnology, and nanorobotics which is defined as the discipline of designing and constructing nanorobots whose components are at or near the scale of a nanometer [36]. Nanorobots have been documented in the literature to be used for acceleration of tooth movement in animal studies through the use of nanoelectromechanical systems (NEMS) [37, 38] and nano LIPUS ultrasound devices [39–41]. Moreover, the concept of a smart bracket with an integrated nanomechanical sensor system for 3D force and moment real-time measurement has shown to work well, allowing precise application of force by an orthodontist [42]. Nanorobotic dentifrice delivered by mouthwash or toothpaste has been proposed for better cleaning of teeth [43]. Not only this but also nanosensors have been tested and validated for objective remote monitoring of removable appliance wear [44–46].

A noteworthy contribution of robotics in orthodontics is their applications in implant placement and maxillofacial surgeries including cleft palate surgeries, improving surgical efficiency and precision [47–49]. In 2017, the dental implant navigation robot system manufactured by Neocis Inc, called Yomi, received FDA approval and became the world's first commercially available oral implant robot [50].

The remarkable increase in the number of patients seeking aligners for treatment has led to a flourish of different aligner companies. The ability to consistently fabricate dimensionally accurate, custom-made, and removable orthodontic appliances in large quantities is a manufacturing challenge that has only recently been met through advances in scanning and automation technology. Align Technology uses stereolithography technology to create its reference models. These SLA resin models are loaded into an automated aligner-forming system that heats, forms, and laser-marks sheet plastic over each plastic model. These parts are transported on a conveyor belt to a robotic arm that loads each part into an automated cutting machine for trimming and molds and carves out each custom tray with laser precision [51, 52].

Finally, robots play a vibrant role in the treatment of TMD through massaging robots, mouth opening robots, and neurological rehabilitative exoskeleton robots, promoting active participation of the patient and accurately tracking the progress of a patient over time, by using progressive therapy routines [53–55].

Today's technological advancements allow for more efficient programming schemes for the robots to work in different situations, through the implementation of machine learning (ML) and artificial intelligence (AI). Machine learning involves the different methods for making use of large amounts of data to learn and self-improve from its own experience [56]. As for artificial intelligence in the context of robotics, it is the field of autonomous and symbolic task planning that is used to automatically plan a sequence of actions to reach a specific goal. Furthermore, by artificial intelligence, robots are able to reason about current situations and new events in order to adapt to new circumstances autonomously [57].

How far have the aforementioned robotic applications in orthodontics found applicability, and what are the future research directions that are proposed based on the results of the researches? This scoping review is aimed at mapping the existing technological robotic applications in orthodontics as reported in orthodontic literature in the last decade.

## 2. Methodology

**Figure 1:**
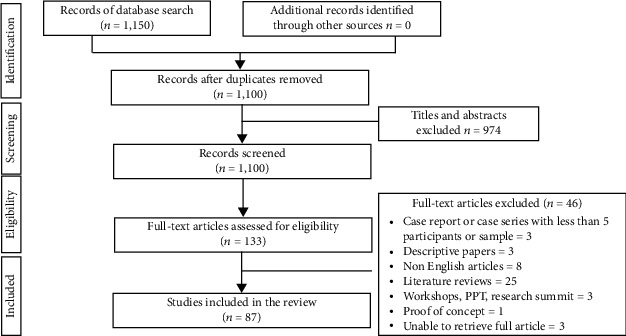
Preferred reporting items for systematic reviews and meta-analyses.

A scoping review of literature was carried out by following the preferred reporting items for systematic reviews and meta-analyses (PRISMA) guidelines (Figure 1). The study protocol was developed to address the main research question and the study's eligibility criteria (Table 1). The scoping review was performed on MEDLINE, Cochrane Library, EMBASE, PubMed, Google Scholar, Web of Science, and Science Direct to collate the studies on robotics in orthodontics. The various search terminologies used are presented in Table 2. The literature search was dated back to ten years from the time of this review and was limited to English language only (January 2010 to January 2021). Meline [58] had suggested that the time period for the search of “contemporary studies” may be limited to ten years in order to maintain relevancy. IRB was obtained from the European University, DHCC, Dubai, UAE.IRB:EUC/67/2020/54.

The primary research question, “robotics in orthodontics,” was further subcategorized into eight domains, as shown below:

1. Robotic dental assistants
2. Robotics in diagnosis, management, and simulation of orthodontic problems
3. Robotics in orthodontic patient education, teaching, and training
4. Wire bending robotics including labial and lingual wire bending robotic systems and customized fixed appliance robotics
5. Nanorobots/microrobots for acceleration of tooth movement and for remote monitoring and telecommunication
6. Robotics in maxillofacial surgeries and implant placement
7. Robotics in automated aligner production
8. Rehabilitative robots in management of TMD

“PICO” guidelines were followed to charter the full-text analysis and data extraction of the identified original research. The data included the first author and year of publication, study design, number of participants, intervention, comparison, outcome (primary and secondary), method of measurement, and the domain tested (Supplement 1).

**Figure 2:**
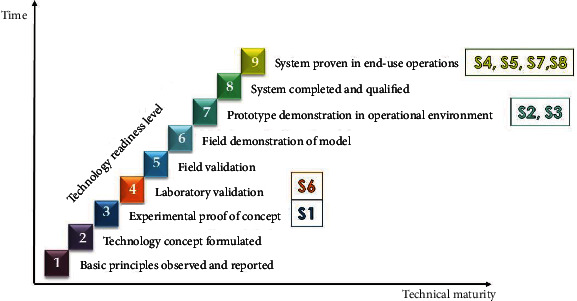
The technological advance of a system may be described using the technological readiness level (TRL 1-9) introduced by Mankins in 1995 [59]. Different robotic applications in orthodontics assigned to different technological readiness levels.

The different presented robotic technologies in orthodontics were assigned a technological readiness level depending on the information provided from the publications [59] (Figure 2). Level 1 was assigned when a basic description of a system principle is observed and reported. When a full concept for a system was formulated, level 2 was assigned. Levels 3 and 4 included in vitro validation, whereas levels 5 and 6 were field validations. When a prototype is demonstrated in the operating environment, technological readiness level 7 is achieved. Level 8 is reserved for qualified complete systems, and level 9 is reserved for when a system has been proven in end-use operations. Furthermore, the application areas were put together in a year-wise categorization in order to point out the advances within the different fields.

## 3. Results

The initial database and additional search resulted in 1,150 records, of which 133 relevant articles were retrieved in full. 87 studies met the selection criteria following screening and were included in the scoping review (Figure 1).

The studies that were included in the review and excluded studies with reasons are enumerated as supplements to this article.

**Figure 3:**
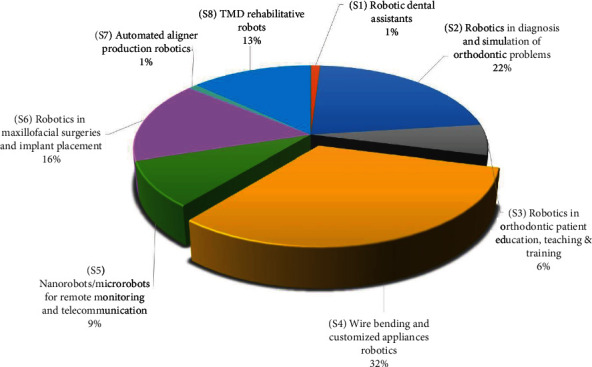
Pie chart showing percentage representation of eight domains of robot applications in orthodontics.

Robotic wire bending and customized appliances have the largest share with 32%, followed by 22% share by diagnosis and orthodontic simulation using robots, followed by 16% share by robotic use in maxillofacial surgeries, robotic uses in TMD management by 13%, and nanorobotics and telemonitoring by 9%. The least represented were robotic use in orthodontic teaching and education, robotic assistants, and robotic automated aligner production representing only 6%, 1%, and 1%, respectively (Figure 3).

According to the technology readiness level reached of different domains, wire bending robots, nanorobots, TMD robots, and automated aligner productive robots reached the highest level of technological readiness [9]. Robots used in diagnosis and patient robots reached level [7] of technological readiness, whereas surgical robots and assistive robots reached only level [4] and level [3] of technological readiness, respectively (Figure 2).

Upon reviewing the scope of the published literature in the use of robotics in orthodontics over the last decade, 8 main domains can be subcategorized in which robotics could have an application in the orthodontic specialty.

### 3.1. Robotic Dental Assistants

A prototypical 7DoF robot assistant was proposed by Grischke et al., in 2019. The authors investigated the possibility of active robotic support during treatments by handling of instruments via a multimodal communication framework that is aimed at dentists as users. The users almost reached expert level time after only a short overall interaction time, with the visual gestures being the most difficult to handle, while the web interface and verbal and haptic gestures were more robust, demonstrating usability and feasibility beyond a controlled experimental setup [60].

### 3.2. Robotics in Diagnosis, Management, and Simulation of Stomatognathic System and Orthodontic Problems

#### 3.2.1. Parallel Robot for Dental Articulation

An optimal parallel robot for dental articulation is able to not only solve the traditional problem in dentistry but also to eradicate the technical difficulty of duplicating the positions and motions of the patient jaw in dental practice, reducing dentists' chairside time greatly and increasing the efficacy of dental workflow by reducing the traditional trial-error approach [17].

#### 3.2.2. Robot for X-Ray Imaging

In 2013, a robot equipped with a skull to investigate the influence of head movement to the accuracy of 3D imaging was proposed [61].

#### 3.2.3. Automated Cephalometric Landmark Annotation

A new approach for automatic 3D cephalometric annotation using shadowed 2D image-based machine learning was proposed to overcome the existing serious difficulties in handling high-dimensional 3D CT data, achieving an average point-to-point error of 1.5 mm for seven major landmarks [62]. Also, another study using a patch-based iterative network with a three-layer CNN architecture for automatic landmarking of a CT image showed that landmarks can be automatically calculated in 37.871 seconds with an average acceptable accuracy of 5.785 mm [63].

#### 3.2.4. Robots for Remotely Controlled Mandibular Positioners for Obstructive Sleep Apnea Patients

The concept of a remotely controlled mandibular positioner (RCMP) for single-night titration was introduced to determine an effective target protrusive position (ETPP) for every individual patient within 45 minutes [64], to prospectively predict treatment success and outcome to avoid a titration procedure for weeks or months and to identify favorable candidates for oral appliance therapy [65–67]. The RCMP consists of a controller that receives commands from the device software and, in turn, activates a stepping motor attached to dental trays in the patient's mouth [64].

#### 3.2.5. Robotic Approach to the Reduction of Dental Anxiety in Children

Robotic technology was successfully found to help in the management of younger children by helping them to cope with dental anxiety and stress, making them behave better in the dental office, through the use of robotic technopsychological distraction techniques [68].

#### 3.2.6. Software Simulation System for Dental Orthodontic Robot

Designing a software simulation system for a dental orthodontic robot was proposed by using Blender's secondary development technology, which transforms orthodontic surgery into a simulation operation that can be interactively simulated in a computer through tooth arrangement algorithms and calculations of tooth positions. Also, it can design orthodontic brackets suitable for patients with dentition malformation [69].

#### 3.2.7. Bionic Robots for Simulation of Stomatognathic System

*Masticatory chewing robots* have been designed, some with a hybrid neural network approach for kinematic modeling, to reproduce human chewing behavior, cycles, chewing forces acting on teeth, jaw dynamic movements, and reactive forces on the TMJ as well as specifying different chewing patterns [11, 12, 70–74].

*Bionic Jaw Motion robots* registering and reproducing mandibular movements were investigated. It is based on two components: a jaw movement analyzer and a robotic device that is able to accurately reproduce recorded movements with no mathematical transformations, reducing mechanical tolerances and time as fast as 5 to 10 s [14].

A *tongue soft robot* which can mimic a few movements of the human tongue was designed with a series of embedded chambers using a pneumatic actuation pattern [13].

### 3.3. Robotics in Orthodontic Patient Education, Teaching, and Training

A humanoid, a full-body patient simulation system (SIMROID), was tested in 2018 among dental students to find out whether a robotic patient was more realistic for the students to familiarize with real patients than the usually used dummies. Students recognized the educational value of the robot patient especially for “risk management” [75]. A patient robot for practicing orthodontic bonding was introduced suggesting that it is useful in orthodontic bonding practice by providing immediate feedback after training and iterative learning [76].

Also, a medical emergency robot was introduced with the aim of helping dental students to get familiar with emergency situations [77, 78]. Another robotic educational equipment described in the literature is the ROBOTUTOR to demonstrate tooth-cleaning techniques to patients. It was found to be the most attractive method (according to patient evaluation) for dental health care education compared to other methods (clinician or video audio tutorial) [79].

### 3.4. Wire Bending and Customized CAD/CAM Appliance Robotics

Accurate arch wire bending is a key technology for fixed orthodontic treatment [80]. Compared with the traditional manual bending system, the accuracy and efficiency of arch wire bending can be improved by using the robot with its precise posture control ability [81].

Different types of arch wire bending robots have been proposed in the last decade including the Motoman UP6 robot, optimizing bending process and properties [80, 82–84], LAMDA system (Lingual Arch wire Manufacturing and Design Aid), bending only 1st-order bends in the *XY* plane [29], motion planning and synchronized control of the dental arch generator of the multimanipulator automatic tooth arrangement robot [85, 86], Cartesian type arch wire bending robot using the third-order *S* addition and subtraction curve control method of the motor to bend the arch wire [81, 87], an end effector for arch wire bending robot that could change the pincer automatically, as needed [88], wire bending robots considering the slip warping phenomenon that exists during wire bending, thus compensating for the spring back of the arch wire [89–91] and greatly improving the bending precision, and robots using the Bessel curve to carry out the control point planning and angle planning showing practicality in clinical treatment [92]. Different descriptive papers analyzed the structural dynamics of bending robots [93] and their various elements [80, 94], verifying the feasibility of the manufacture strategies of formed orthodontic wire fulfilled by different wire bending robotic systems. Additionally, the performance of a more accurate and reliable method of shape setting of superelastic Nitinol tubes has been validated [95].

Moving on to the customized CAD/CAM full appliances including customized brackets and wires manufactured by robots, clinical outcomes were assessed in terms of effectiveness and efficiency in different CAD/CAM systems in comparison to conventional approaches, showing premise in improving or at least achieving similar outcomes to conventional appliances [26, 31, 33, 34, 96]; also, it can reduce overall treatment duration [26, 32, 96, 97].

Upon comparing lingual (Incognito) and labial (Insignia) appliances, it was found that Incognito was more efficacious [28]. In addition, the precision of virtual setup implementation was found to be clinically successful, achieving tooth movement as planned in the setup, with custom arch wires fabricated by different CAD/CAM appliances [27, 97–100], though the accuracy differs with the type of tooth and movement [27].

### 3.5. Nano-/Microrobots

#### 3.5.1. Nanorobotic Dentifrice (Dentifrobots)

Subocclusal dwelling nanorobotic dentifrice delivered by mouthwash or toothpaste could patrol all supragingival and subgingival surfaces, performing continuous calculus debridement. These invisibly small dentifrobots would be inexpensive, safely deactivating themselves if swallowed, and would be programmed for better cleaning of the teeth [43].

#### 3.5.2. Nanosensors for Remote Monitoring of Removable Appliance Wear

*[1] Monitoring of Obstructive Sleep Apnea Oral Appliance Compliance*. Sleep apnea monitoring devices are being developed for diagnostic and treatment applications as well as monitoring applications. These can be a safe, reliable, effective, feasible, and affordable option to monitor a person's sleeping patterns and to objectively measure compliance in wearing the OSA oral appliances [45, 101].

*[2] Monitoring of Compliance of Active and Passive Removable Appliance Wear*. Compliance in removable appliance wear is a highly variable, multifactorial issue that requires objective measures to be safely addressed in research designs and in clinical practice [44, 102, 103]. Electronic microsensors, such as the Smart Retainer [104] and the TheraMon [46, 102, 105], seem quite promising since they are easy to use and because they have been proved reliable and accurate enough to measure wear time of removable orthodontic appliances by identifying temperature changes, which are then transformed to wear time information.

They are helpful in modifying patients' motivation and determining the extent of impact of current therapeutic procedures and/or patients' cooperation on the treatment outcome. Moreover, they provide the basis for more individualized wear time recommendations for patients with removable appliances, resulting in a more efficient, shorter, and less painful orthodontic therapy [105].

### 3.6. Robotics for Implant Placement and Maxillofacial Surgeries

#### 3.6.1. Robots in Implantology

Robots can be used to measure forces and stresses on the implant and between implant and bone and measure the torque and stability to drill the implant site, as well as to place the implant inside the bone with improved accuracy during operation. Different systems include preoperative planning software, surgical robotic arms, stress sensors, coordinate measuring machines, and optical navigators. The systems consist of preoperative and intraoperative stages. The preoperative stage uses the 3D views obtained from the raw images of the patient before surgery followed by the intraoperative stage, which shows 3D orientation of surgical instrument position and trajectories which are displayed on the monitor within a patient's 3D imaging data [106–110].

#### 3.6.2. Robots in Maxillofacial Surgeries

A lot of systems have been proposed comprising surgical robots with optical surgical navigation systems and some kinds of hard tissue lasers that are able to automatically perform an osteotomy operation according to a preformed surgical plan. During the operation, the robot is proposed to register patient movements by real-time tracking. Robotic surgical techniques are being used for milling of bone surfaces, drilling of holes, deep sawing osteotomy cuts, selecting osteosynthesis plates, bending and intraoperative positioning in a defined position, and orthognathic surgery planning [111–117].

Also, the use of a robot for cleft palate repair has attracted the attention of scholars, and preliminary research results have been achieved, improving surgical efficiency and precision while reducing potential secondary injury to the patient as it reduces the damage to the blood vessels, nerves of the palate muscles, and mucous membranes [118, 119].

### 3.7. Robotics in Automated Aligner Production

In 2011, Hilliard patented a robotic system for forming features in orthodontic aligners, including a control system, a platen for three-dimensional positioning of the aligner, a heating station for selectively heating a small region of the aligner, and a thermoforming station for manipulating the heated region to form a desired feature in the aligner. The control system can include a processor with CAD software to enable a user to design features for aligners. The present invention enables an automated process for installing activation features and other types of features needed for polymeric shell orthodontic aligners to receive auxiliary devices that serve to expand their usefulness, range, and duration of application [120].

### 3.8. Rehabilitative Robots in Management of TMD

Massaging robots and mouth training robots [121, 122] have been proposed for the implementation of safe and effective maxillofacial massage and exercises to treat patients with myofascial pain and limited mouth opening by decreasing muscle stiffness significantly. Suitable treatment regimens have been discussed and evaluated, reaching an efficacy of 70.3% [123], with sonographic features, as the frequency of visibility of the distinct echogenic bands the elasticity index ratios, being a guiding predictor of therapeutic efficacy [124].

Moreover, neurological rehabilitative exoskeleton robots have been introduced for the practical rehabilitation of patients with TMD [125, 126]. Different designs were suggested including the use of a soft pneumatic actuator allowing comfortable patient interaction [127]; a shoulder-mounted robotic exoskeleton for better esthetics and portability, incorporating visual feedback into therapy routines to promote active participation with safety design considerations [128]; assisted motion of the jaw using EMG- and ECG-based feedback systems accurately tracking the progress of a patient over time [129]; and central path generator concept for real-time online trajectory generation, allowing the adaption to the environment and changing the chewing pattern in real-time parameters in a smooth and continuous manner [130, 131].

## 4. Discussion

The scoping review on robotic applications in orthodontics was conducted to evaluate and quantify published orthodontic literature that evaluates uses of robots in orthodontics. A scoping review aids to map the broad outcomes and invention utilization and collates the range of study designs and methodologies implemented. The research question, “What are the uses of robotics in orthodontics?,” was divided into eight main domains, and each was addressed with the PICO framework for literature evaluation.

The greatest representation in literature (32%) was by robotic arch wire bending and customized CAD/CAM appliances. It is an anticipated outcome that robotic wire bending and CAD/CAM appliances are the most frequently measured domain because orthodontics since its inception has always tried to improve the efficacy and efficiency of appliances through the change and improvement of many modalities over time from 3D-assisted diagnosis and management to customized CAD/CAM appliances with automated wire bending robots. The use of a robot to bend an arch wire was found to be a rapid, reliable, and reproducible process that increases the treatment accuracy, efficacy, and efficiency when compared to conventional arch wire manufacturing, reducing the treatment time and the patient discomfort [25, 47, 132, 133]. Likewise, using customized brackets manufactured by robots, the treatment effectiveness and efficiency can be improved by overcoming individual tooth morphology variations and by precise virtual planning of individual tooth movements [26, 31–34, 96, 98–100]. This was reflected in the highest level of technological readiness (9) in which the systems were proven in end-use operations [96, 98–100].

The next domain that received significant consideration (22%) is diagnosis and simulation of orthodontic problems using robots. Reaching a correct diagnosis has been always considered the most crucial step in the orthodontic journey. Diagnosis has witnessed dramatic breakthroughs in the last decade with the advent of the digital era that enabled clinicians to use the best available data for evidence-based diagnosis, treatment planning, and execution of treatment. Simulation of the patient stomatognathic structures including masticatory muscles, tongue, and mandibular and condylar movements in real can help the clinicians to better visualize the problem in 3D, making it easier to reach a correct diagnosis [13, 14, 72]. Robots for dental articulation can eradicate technical difficulties of duplication positions and motions encountered in the classical articulator, thus saving time and producing more precise occlusal relationships [17]. Robots for automated cephalometric landmarking save time and decrease the dependence on professional experience [62, 63]. Titration of oral appliances for treatment of OSA patients in a single night within minutes was introduced by robots to overcome the time-consuming trial-and-error procedures. This can help diagnose favorable candidates for oral therapy as well as predict treatment success [64–66]. Though diagnostic robots gained significant consideration in the literature, it is still in level (7) of technological readiness.

Interestingly, 16% share of outcomes reported is on the use of robots in implantology and maxillofacial surgeries. The anatomy of the oral and maxillofacial region is complex, and the esthetic demand in this dental field is high. Therefore, orthognathic surgery needs to be highly accurate and performed with minimal trauma, and this can be accomplished using robotic surgeries. There are systems providing haptic navigated robot technology to provide physical guidance on the position, orientation, and depth of the drill, moreover allowing the practitioners to visualize the surgical site and to register patient movements by real-time tracking, enabling them to change the plan in real time according to the specific circumstances during surgery [50]. Robots provide accuracy in measuring torque values and insertion depth in a more precise manner in mini-implant placement. This minor surgical procedure done by robots reduces the chances of failure of mini-implant placement, saves time, and reduces the risk of infection [134]. Unfortunately, the invasive character of surgeries may impair the acceptance of this technology among patients and orthodontists. Hence, these most invasive applications are not very suitable as forerunners, reaching only level (4) of technological readiness.

Although rehabilitative robots for TMD (13%) and nano-/microrobots for remote monitoring of compliance of removable appliance wear (9%) came in the 4^th^ and 5^th^ places, they are showing a great premise in the orthodontic research, evidenced by the highest technological readiness level reached (9). Different rehabilitative robots for management of TMD have been proposed with well-established protocols, promoting active participation of the patient and accurately tracking the progress of a patient over time, by using progressive therapy routines with smooth continuous transitions between movements, increasing patient comfort [128, 130, 131].

In the same ways that technology research and development drove the space race and nuclear arm race, a race for nanorobots is occurring. The reasons behind this are that large corporations, such as General Electric and Siemens, have been recently working in the development and research of nanorobots [135]. Surgeons are getting involved and starting to propose ways to apply nanorobots for common medical procedures [136], and universities and research institutes were granted funds by government agencies towards research developing nanodevices for medicine [137].

Nanorobots have been proposed earlier for the acceleration of tooth movement which is always a matter of great concern to both orthodontists and patients. This could be done through the induction of electrical or ultrasound waves which enhance cellular enzymatic phosphorylation activities and fibroblast growth factor release from a macrophage-like cell line (U937), accelerating tooth movement [37–41]. The idea of incorporating microrobotic sensor systems to control the 3D-force-moment of the orthodontic bracket was enormously promising in nature; however, the innovation is yet to test the telemetric energy and data transfer phase [42]. Nanorobots for remote monitoring of compliance is proven to be a safe, reliable, feasible, and more accurate objective evaluation of the compliance and effectiveness of treatment over their subjective alternatives of evaluations including questionnaires and patients reporting of wearing hours. Moreover, they provide the basis for more individualized wear time recommendations for patients with removable appliances, resulting in a more efficient, shorter, and less painful orthodontic therapy [44, 45, 105]. It has to be unequivocally acknowledged that robotic objective monitoring will be an important determinant of orthodontic care protocols in the future.

Research on educational robotics (6%) in university environments seems to be an appealing initiator to introduce robotics and take the first hurdle towards acceptance of robots among future orthodontists, as it has already reached a reasonable level of technological readiness (7). “Hanako,” the SIMROID robotic patient, is a real contribution to the educational terrain. It is standing 165 cm tall. It comes with a metal skeleton and vinyl chloride-based gum pattern of the skin. It is imitating a human in its actions and expressions. It can verbally express pain, roll its eyes, blink, shake its head in pain, and perform movements of the jaw, tongue, elbow, and wrist. Furthermore, it can simulate a vomiting reflex with a uvula sensor and also simulate functions to induce bleeding and saliva flow. It provides emotional feedback to dentists especially pain and discomfort and also responds and reacts to questions and commands and finally rating and evaluating their treatment, thus helping the dental students to learn in a better way [20].

The least addressed domains were the automated aligner production robots and the assistive robots, accounting for only 1% both. Although a lot of research has focused, during the last decade, on the effectiveness and efficiency of the use of aligners in controlling tooth movement and treating different malocclusions reaching the highest level of technological readiness (9) [138, 139], very few of them tackled the importance of the role of robots in their fabrication and in the automated attachment fabrication [120]. Likewise, research in the field of assistive robotics, though not much with the lowest technological readiness level (3), seems to be promising to facilitate the introduction of this new robotic enabled era, since robots will help keep the records of the patients, manage the appointments, and assist the orthodontist without tiring.

As observed, the percentage of different domains as a reported outcome was not always positively correlated to the level of technological readiness reached. To be more specific, wire bending robots, TMD robots, and nanorobots gained both significant contribution as reported domains and also reached the highest levels of technological readiness [9], whereas diagnostic robots and surgical robots gained a high percentage as reported domains but lower levels of technological readiness (7 and 4, respectively). A patient robot was a moderately reported domain and at the same time reached a moderate level of readiness [7]. An assistive robot was both lowest as a reported outcome and in technological readiness level [3]. Despite the fact that automated robotic aligner production was one of the least reported domains, it reached the highest technological readiness [9], due to the growing market of aligner companies, as well as the remarkable increase in patients seeking more esthetic alternatives of appliances.

A reasonable explanation why robotics is still a field of low interest in dentistry may be the lack of expert knowledge to program and control those systems as a nonprofessional. Consequently, research in this domain still relies on efficient collaboration between engineers and dentists. This may rapidly change in the near future as the robotic community researches novel programming paradigms and interaction methodologies in order to make communication between robots and humans as intuitive as possible. Furthermore, the use of ML and AI methods to autonomously plan tasks and reason about the environment may further reduce the effort on the user side when using a robot.

A noteworthy point to mention is the tremendous effort that is required of orthodontists and dental assistants in order to learn to work with these advanced technologies. Older generations may be more used to familiar tools and are rather skeptical to adapt. However, new generations of orthodontists can be considered digital natives, and their experience might lead them to use digital tools more naturally. Moreover, in light of the expected developments in robotics, AI and ML, future generations may even be considered “robonatives” [140].

All the aforementioned robot applications presented in this review have an inherent potential to advance dentistry far beyond digitalization and into a new world where digitalization reaches out to manipulate our real world. However, the overall technological readiness is still low, and more effort and research are needed for optimum utilization of the real value of robotics. On the other hand, there are numerous approaches in the research community to explore the potentials and challenges of integrating robotics, AI and ML, into dentistry; thus, the speed of innovation in this novel field should increase in the upcoming years. Knowledge gaps identified could be core outcome sets (COS) for scholarly literature in the future (Table 3).

Our world is witnessing exceptional events over the past two years of the COVID-19 pandemic, a new era that will be a significant turning point in the world's history. Despite our accumulated experience in crisis management, this virus has been able to isolate us all in our homes. With the emerging robotic technology, orthodontists can easily practice orthodontics remotely with its numerous applications in the different domains in this life-threatening pandemic, following the new norms of social distancing and reducing the human working hours, thus dramatically decreasing the risk of infection to the orthodontists as well as to the patients.

## 5. Orthodontic Applications of Robotics: Crystal Gazing into the Future!

1. Larger scale, human clinical trials need to be done to test the feasibility, safety, accuracy, and usability of different robotic systems
2. 4D printing of soft robotics can replicate natural physiomechanical changes over time leading the transition from static to dynamic, with precise controllability and unlimited reversible actuation [141]
3. Machine learning methods and artificial intelligence are used to train robots to reliably perform their assigned tasks and even to be able to reason about current events and new information in order to adapt to new situations
4. The flexibility and motor functions of robotic arms need further technical advancements to suit different individual clinical situations
5. For arch wire bending robots, the research in the future needs to focus on the arch wire spring back and bending algorithm, adapting bending to more complicated clinical arch wires, as well as improvement of plier design for dexterous collision avoidance [86]
6. Friendly human-computer interaction software is designed to provide humanization input and feedback for the operators
7. Advanced self-conscious robot control by patients using surface EMG (sEMG) signal of the facial muscles is developed to guide the actuation of the robot [127]
8. Further prospective studies with larger numbers of patients and longer follow-up periods are required to confirm the success and the evolution of OA compliance patterns over time [45]
9. Orthodontic material testing is done by robots

## 6. Conclusions

The orthodontic specialty is moving forward towards a new era of data-driven and robot-assisted medicine. Robotics is by all means a breakthrough in the field of technology, and its evident applications in orthodontics are potentially immense. Noteworthy, with the incorporation of AI and ML to our day-to-day clinical practice, there are speedy improvements in precision and success of our treatments through the implementation of robots. Hence, it is very important for all clinicians to have basic knowledge and training with these technologies. However, the latest step changes in modern robot technology, ML and AI, have not yet been fully introduced to orthodontic research nor have they reached technological readiness and cost-efficiency to enter the dental market.

Arch wire bending robots, simulative robots for diagnosis, and surgical robots have been important areas of research in the last decade. Rehabilitative robots and nanorobots are quite promising and have been considerably reported in the orthodontic literature. On the other hand, assistive robots, patient robots, and automated aligner production robots need more scientific data to be gathered in the future.

In fact, the increased intuitiveness of the systems combined with broad educational efforts and introduction of affordable systems are key challenges that need to be overcome to truly introduce robotics to orthodontics.

## Figures and Tables

**Table 1:** Scoping review selection criteria.

Inclusion criteria	Exclusion criteria
Studies including randomized controlled trials (RCTs), controlled clinical trials (CCTs), cohort studies, retrospective studies, and case-control studies on the eight subcategories as enumerated in the text. Descriptive technique article (focused on design description). Any type of comparison with conventional mode of orthodontic treatment, method, or approach. All types of reported outcomes (primary and secondary).	Case reports and studies with less than five participants or sample. Personal opinions, narrative articles, letters to editors, or interviews. Proceedings from research summits. Systematic reviews, meta-analyses, and review articles. Proof of concept, workshops, and presentations.

**Table 2:** Table depicting the search terminologies employed for the scoping review.

S/N	Database	Search term 1 (main search term)	Search term 2	Not relevant
S1	(2010-2021 full text, English) MEDLINE, Cochrane Library, EMBASE, PubMed, Google Scholar, Science Direct, Web of Science	Robotic dental assistant		(1) Letters to editors, opinion articles, descriptive papers, interviews (2) Systematic reviews, meta-analyses, and review articles (3) Proof of concept, workshops, presentations (4) Proceedings from research summits (5) Case reports and studies with less than five participants or samples
S2		Robotic orthodontic diagnosis	Robotic simulation of orthodontic problem/masticatory/chewing/tongue/mandibular movement/condylar movement robots/articulation Robotic X-ray/robotic cephalometric landmarking/robotic management of anxiety	
S3		Robotic orthodontic teaching, education, training	Robotic patient	
S4		Orthodontic wire bending robotics	Customized orthodontic wires/customized appliances/customized brackets Sure Smile, Incognito, Insignia, BRIUS, LAMDA	
S5		Nanorobotics/microrobotics	Remote/telemonitoring/telecommunication/smart brackets/objective measurement of compliance/acceleration of tooth movement	
S6		Robot orthognathic surgeries	Robot maxillofacial/implant surgeries/mini-implant placement	
S7		Robotics in aligner production	Automated aligner/automated attachment	
S8		Robots in TMD	Rehabilitative robots, massage robots, mouth opening robots, neurological rehabilitative robots	

S1 (search 1): robotic dental assistant; S2 (search 2): robotics in diagnosis, management, and simulation of orthodontic problem; S3 (search 3): robotics in orthodontic patient education, teaching, and training; S4 (search 4): wire bending robotics including labial and lingual wire bending robotic systems and customized fixed appliance robotics; S5 (search 5): nano-/microrobots for acceleration of tooth movement and for remote monitoring; S6 (search 6): robots in maxillofacial surgeries and implant placement; S7 (search 7): robotics in automated aligner production; S8 (search 8): rehabilitative robots in management of TMD.

**Table 3:** Knowledge gaps identified which could be core outcome sets (COS) for scholarly literature in the future.

(1) Prospective human trials assessing biocompatibility, efficacy, efficiency, and cost benefit ratio of robotic systems
(2) Clinical audit of 4D printing applications
(3) Clinical audit of AI-based robotic training
(4) Tactile and motor movements of robotic arms
(5) Clinical efficacy of advanced wire bending mechanics
(6) Performance of human-computer interface to be tested in clinical situations
(7) Surface trackers guided robotic movements
(8) Orthodontic material evaluation for precision in material science

## Data Availability

The data used to support the findings of this study are included within the article.

## Supplementary Materials

Supplementary Materials Supplement 1: studies included for the scoping review (*n* = 87). Supplement 2. studies excluded from the scoping review (*n* = 46) [7, 29, 30, 35, 37, 38, 43, 47, 48, 132, 133, 142–176].
