# Supplementary material for: Robotic Applications in Orthodontics: Changing the Face of Contemporary Clinical Care
Source: Biomed Res Int. 2021 Jun 16;2021:9954615. doi: 10.1155/2021/9954615 (PMC8225419; doi:10.1155/2021/9954615)
Supplement: Supplementary Materials — Supplement 1: studies included for the scoping review (n = 87). Supplement 2. studies excluded from the scoping review (n = 46) [7, 29, 30, 35, 37, 38, 43, 47, 48, 132, 133, 142–176]. [file 9954615.f1.docx]

**Supplement 1. Studies included for the scoping review (n = 87).**

| **Sr. No** | **Author /Year** | **Study Design** | **Participant** | **Intervention** | **Comparison** | **Outcome Primary/ Secondary** | **Method of measurement** | **Domain** |
| --- | --- | --- | --- | --- | --- | --- | --- | --- |
|  | Grischke, 2019^(^[^60^](#_ENREF_60)^)^ | First concepts and pilot controlled experimental setup user study | 20 participants | Collaborative robots in dental assistance | Real assistants | 1. Understand the effect between experience, usability and feasibility of assistive robots 2. Find the most frequently used interaction modalities | Survey | S1 |
|  | Chang, 2012^(^[^11^](#_ENREF_11)^)^ | Descriptive technique | Masticatory robotic model | Mastication robot of lead screw and scotch-yoke actuation | Actual masticatory process and jaw dynamic movements | 1. Evaluate prototype success of simulation of jaw movements 2. Evaluate output force relative to input energy | Mastication robot moving trajectory measurements by system mathematical models | S2 |
|  | Remmers, 2013 ^(65)^ | A prospective, blinded, outcome study | 67 patients recruited from a sleep center or a dental practice  age 21-80 years | Prediction of therapeutic outcome with a mandibular protruding oral appliance using a remotely controlled positioner | Polysomnographic study | 1. Accuracy of standard predictive parameters (sensitivity, specificity, positive and negative predictive values) 2. Efficacy of achieving effective target protrusive position | 1. Portable monitor 2. PSG monitoring by a computerized system 2007 3. American Academy of Sleep Medicine manual for scoring of sleep and associated events | S2 |
|  | R Spin-Neto, 2013^(61)^ |  | Fully dentate human skull incorporated into a robot | CBCT image artefacts related to head motion simulated by a robot skull | No motion as reference | Assess artefacts and their impact on CBCT image quality after head motion simulated by a robot skull | 1. Qualitative assessment of Axial images 2. A 100 mm visual analogue scale (VAS) was used to quantitatively assess image quality | S2 |
|  | Lu X,  2015^(^[^13^](#_ENREF_13)^)^ | Descriptive technique | Soft robotic model | Soft robot mimicking human tongue | Real tongue movements | Assess success of simulation of different tongue movements relationship between deformation range and structural parameters | Finite element method simulations carried out using software Abaqus | S2 |
|  | Kalani H, 2015^(^[^12^](#_ENREF_12)^)^ | Descriptive technique | 1. 6-UPS parallel mechanism mastication robot model 2. 3 male subjects (age range: 24–28 years) | A hybrid neural network approach for kinematic modeling of a novel 6-UPS parallel human-like mastication robot | Process of solution-finding for direct kinematic problem of the conventional Newton Raphson  method | Assess accuracy, the number of iterations and computational time  for the human like mastication robot motion | 1. Number of iterations, the maximum allowed error, and computational time 2. Vector algebra to obtain its direct and inverse kinematic formulations | S2 |
|  | Sutherland, 2017^(66)^ | Clinical sleep laboratory setting | 40 adults with OSA  57.1 ± 11.6 y | RCMP method for oral appliance treatment outcome prediction in OSA | Standard overnight polysomnography with the treatment oral appliance  in situ | Validation and accuracy assessment of RCMP as a prediction technique for oral appliance treatment outcome | 1. Device software (OATRx TS) 2. American Academy of Sleep Medicine scoring rules 3. Prediction algorithm from the original RCMP study | S2 |
|  | Kastoer, 2018^(^[^64^](#_ENREF_64)^)^ | Prospective  clinical trial | Ten patients in whom OSA was diagnosed  age 54 ± 9.5 years | Remotely controlled mandibular positioner for the determination of effective target protrusive position | Will be done in future research in comparison to polysomnography | Assess feasibility, protocol and timing of RCMP | 1. RCMP-controlled man-operated software. 2. Fiberoptic nasendoscope (Type ENF-GP, Olympus) | S2 |
|  | Lee, 2019 ^(^[^62^](#_ENREF_65)^)^ | Descriptive Technique | 27 anonymized CT datasets generating  2,700 shadowed 2D images | Automatic 3D cephalometric annotation system using shadowed 2D image- based machine learning using VGG-net | Manual landmarking of 7 landmarks | Assess accuracy of automatic 3D cephalometric annotation system using shadowed 2D image- based machine learning using VGG-net | Point to point error between tested landmark and the reference landmark using  SimPlant software | S2 |
|  | Mostashiri, 2019^(70)^ | Descriptive technique | Redundantly actuated parallel chewing robot | Measurement of reaction forces on TMJ by redundantly actuated parallel chewing robot | Other mastication robots like 6-DOF chewing robots | 1. Invitro measurement of reaction forces in TMJ during chewing cycles of the robot 2. Provide a robotic platform to evaluate the role of food texture on TMDs | Strain gauges, as force sensors, are added to chewing robot | S2 |
|  | Dieltjens,2019 ^(^[^67^](#_ENREF_67)^)^ | Protocol for prospective randomized crossover trial | 78 patients  with moderate to severe OSA  Age > 18 years | Remotely controlled mandibular positioning of oral appliance therapy during DISE-assisted titration and PSG-guided titration | Conventional subjective titration | 1. Primary outcome: actual mandibular protrusive position found to be the most optimal for each tested titration procedure 2. Secondary outcome: therapeutic efficacy comparison among the different titration modalities | 1. Uniform upper airway scoring system 2. RCMP ruler 3. Software 4. Level 1 sleep study 5. A temperature-sensitive microsensor with on-chip integrated read-out electronics 6. Structured questionnaires | S2 |
|  | Zhou,  2019^(^[^69^](#_ENREF_68)^)^ | Descriptive technique | CT scan models of the patient's oral cavity | Software system of dental orthodontic robot which can simulate dental orthodontics | Traditional orthodontic surgery in the past and domestic dental repair assistant software systems | 1. Assess the simulation of the orthodontic process 2. Designing of orthodontic brackets suitable for patients with dentition malformation | Software system by Blender secondary development technology | S2 |
|  | Kizghin, 2019^(^[^17^](#_ENREF_17)^)^ | Descriptive technique | Robotic articulator model | Robotic articulator suitable for reproducing tracked movements of the patient’s jaw by a tracking system | Conventional dental articulator | 1. Fabrication and testing of robot prototype 2. Evaluate chairside time and the efficacy of dental workflow | MATLAB software | S2 |
|  | Ma, 2020^(63)^ | Descriptive technique | CT images from 66 patients who underwent oral and maxillofacial surgery | Automatic 3D landmarking model using patch-based deep neural networks for CT image | Traditional landmarking method | Assess processing time and accuracy of automated 3D landmarking | MIMICS and MATLAB softwares | S2 |
|  | Mostashiri, 2020^(71)^ | Descriptive technique | Disturbance observer-based controller of a chewing robot | Disturbance observer-based controller for  mimicking a human subject’s mandibular motion by the chewing robot | Mandibular motion of a human subject was recorded to be used as the reference trajectory | In-vitro measurement of the TMJs reaction forces while chewing peanuts and gum. | 1. Portable fiducial-marker- based motion-capture system, PFMS 2. LabView software 3. Force sensors on TMJs of the chewing robot | S2 |
|  | Babu,  2020^(72)^ | Descriptive technique | Masticatory robot model | Development of masticatory robot capable of measuring the forces and moments acting on teeth during mastication | Real masticatory process | Design and development of a masticatory robot to measure the forces and moments acting on teeth during mastication. | 1. Piezoelectric film bed 2. Load cells 3. LabView software | S2 |
|  | Carossa, 2020^(^[^14^](#_ENREF_14)^)^ | Descriptive technique | 1. Bionic jaw motion composed of a jaw movement analyzer and a robotic articulator. 2. a volunteer | Individual  mandibular movement registration and reproduction using an opto-electronic jaw movement analyzer and a dedicated robot | 1. Pantographic tracings and articulator setting 2. Other systems like Arcus Digma | 1. To evaluate accuracy and precision in registration and reproduction of mandibular movements in static and dynamic conditions 2. Evaluate advantages of the proposed system 3. Cost and time saving assessment | 1. High frequency filming camera that acquires 140 frames per second 2. Custom designed software | S2 |
|  | Mostashiri, 2020^(73)^ | Descriptive technique | Redundantly actuated parallel robot model | Optimizing the torque distribution of a redundantly actuated parallel robot | Conventional feedback control methods of steady-state tracking of trajectories of chewing robot | To study temporomandibular reaction forces during food chewing | Force sensors attached to TMJs of robot | S2 |
|  | Kasimoglu, 2020^(68)^ | Randomized controlled clinical trial | Two hundred children  (6.5±1.66years) | Robotic approach to the reduction of dental anxiety in children | Control Group (without robot accompaniment) | Evaluate the success rate of the new robotic distraction technique | 1. Questionnaires 2. Parental Corah Dental Anxiety Scale 3. Facial Image Scale (FIS) 4. Physiological pulse rate 5. Frankl Behavior Rating Scale (FBRS) | S2 |
|  | Alemzadeh, 2021^(^[^74^](#_ENREF_74)^)^ | Descriptive technique | 1. Chewing robot with built in humanoid joints 2. 10 healthy adult dentate subjects | Development of a chewing robot with built- in humanoid jaws to simulate mastication | Chewing using human participants | 1. Assess mastication similarity to humans 2. Quantity and measure release rate of Xylitol in salivary solutions and in the gum bolus 3. *Measure saliva flow Rate and Volume Calculations during Chewing* | 1. A sorbitol/xylitol enzymatic kit 2. UV-Vis spectrophotometer 3. Instron 50KN 2580 series Static Load Cell 4. Custom-built bite sensors 5. Microbalance 6. MATLAB software | S2 |
|  | Newby,  2010^(77)^ | Structured clinical examinations | 1. Final year dental students (n = 52) from the University of Melbourne 2. Patient simulator | Robotic realistic simulation training in the management of medical emergencies | Traditional lecture style teaching | Assess student attitudes towards realistic simulation training in management of medical emergencies | Student responses by questionnaire utilizing a 5-point Likert scale | S3 |
|  | Tanzawa, 2012^(75)^ | Objective structured clinical examination | 88 fifth-grade students in the department of Dentistry at Showa University | Robot patient that can reproduce an authentic clinical situation for dental clinical training | Traditional mannequins | 1. Evaluate educational value of the robot patient especially for risk management 2. Evaluate effectiveness of the robot patient in the dental field | 1. Checkpoints on the examiner’s score sheet 2. Questionnaire survey | S3 |
|  | Ahire,  2012^(79)^ | Randomized clinical trial | 150 subjects with history of gingivitis and only mild periodontitis | ROBOTUTOR | Clinician and audio-video aids | Assess efficacy, effectiveness, ease of understanding, attraction and recollection of different modes of dental health education for demonstration of the Bass toothbrushing technique | Questionnaires and investigators | S3 |
|  | Tanzawa, 2013 ^(^[^78^](#_ENREF_78)^)^ | Objective structured clinical examinations (OSCEs) | 98 fifth-grade dental students  at Showa University | Medical emergency education using a robot patient in a dental setting |  | 1. Evaluate student responses to medical emergencies in the dental setting 2. Evaluate the students’ response to the robot educational value | 1. Student scores for different items on checklist 2. Questionnaire on the educational value of the robot patient | S3 |
|  | Futaki, 2016^(76)^ |  | 1. Nine subjects: 5 dentists 2 years after graduation and 4 residents 1 year after graduation 2. Patient robot | Training robot for orthodontic bonding practice | Mannequin | Investigate the usefulness of a patient robot for orthodontic bonding practice | 1. Examiner’s score: using an evaluation sheet 2. Bonding time 3. Bracket position using cavity/abutment preparation grading system, Fair Grader 100 4. Dentist questionnaire | S3 |
|  | Saxe, 2010^(^[^26^](#_ENREF_26)^)^ |  | Study casts of 62 patients whose orthodontic treatment was completed | Sure Smile | *Conventional approach* | To examine the efficiency and effectiveness of the Sure Smile process | American Board of Orthodontists Objective Grading System  (ABO OGS) | S4 |
|  | Du,  2010^(82)^ | Descriptive technique | MOTOMAN UP6 arch wire bending robot | Robotic orthodontic wires bending based on finite point extension method | Manual practice | 1. To propose to use MOTOMAN UP6 for the bending operation 2. Develop the finite point extension method for robotic trajectory planning | MATLAB software | S4 |
|  | Dan Grauer, 2011^(98)^ | Case control study | Dental casts of 94 consecutive patients from 1 practice, were scanned to create digital models,  and their set ups | Fully customized CAD/CAM lingual orthodontic appliances | Final outcome digital model | Evaluate the accuracy of a CAD/CAM lingual orthodontic technique | Superimposition and calculation of individual tooth discrepancies between the setup and actual outcome in terms of a six-degrees-of- freedom rectangular coordinate system | S4 |
|  | Timothy. Alford, 2011^(96)^ |  | Records of 132 non extraction patients | Finishing with Sure Smile method | Conventional finishing with manual wire bending | Compare clinical outcomes between patients finished with the Sure Smile method and those with conventional fixed orthodontic therapy | American Board of Orthodontics (ABO) Cast/Radiographic Evaluation (CRE) | S4 |
|  | Zhang Y, 2012^(^[^83^](#_ENREF_83)^)^ | Descriptive technique | Robotic arch wire bending | Optimizing algorithm of control points planning of arch wire bending forming | Incremental algorithm and finite points derivation algorithm were performed  and the resulted control points of the fitted curves are compared with ideal curves | To determine a set of control points for robotic arch wire bending process through algorithms | 1. Orthogonal tests of variables in two algorithms 2. Area error between the fitted curve and ideal curve 3. Elapsed time calculation program | S4 |
|  | Zhang, 2011^(^[^84^](#_ENREF_84)^)^ | Bending property analysis and experimental study | 1. Domestic stainless-steel wires, β-titanium alloy wires and Australia stainless steel wires 2. Orthodontic arch wire bending robot | Bending properties analysis of nickel steel wires | Different types of wires | To analyze the bending properties of nickel steel wires, including domestic stainless-steel wires, β-titanium alloy wires and Australia stainless steel wires | Finite analysis software MSC. Marc | S4 |
|  | Zhang, 2012^(80)^ | Descriptive technique | 1. Robotic system for arch wire bending 2. A patient with no clinical experiences of orthodontic treatment | Novel robotic system to bend arch wire into desired configuration | Patient’s oral parameters | Analysis, experimentation and feasibility of the robotic system for arch wire bending | Control software of the robotic system for arch wire bending | S4 |
|  | Larson, 2013^(^[^27^](#_ENREF_27)^)^ |  | Posttreatment models of 23 patients treated with Sure Smile | Computer-assisted orthodontic treatment technology to achieve predicted outcomes | Virtual treatment plan models | To evaluate the effectiveness of computer-assisted orthodontic treatment technology to produce the tooth position prescribed by the virtual treatment plan | Digital superimposition of post treatment models on their corresponding virtual treatment plan models | S4 |
|  | Weber,2013^(^[^31^](#_ENREF_31)^)^ | Retrospective study | Conventional group:11 cases  Insignia group:35 cases | Customized Insignia appliance | Modified Roth-prescription  conventional bracket system, Titanium Orthos | To compare the Insignia system to a modified Roth-prescription conventional bracket system, Titanium Orthos in terms of clinical effectiveness and efficiency | 1. Peer Assessment Rating (PAR) system 2. ABO grading system | S4 |
|  | Jiang, 2013 ^(85)^ | Descriptive technique | 1. Multiplanar Tooth arrangement robotic system 2. a 64 years old male patient with slight alveolar absorption | Motion planning and synchronized control of the dental arch generator of the tooth-arrangement robot | Theoretical control values | 1. Validity of the dental arch generator to automatically generate a dental arch to fit a patient according to the patient’s arch parameters. 2. Evaluate positional accuracy, precision, single point error and arc width directions | MATLAB software | S4 |
|  | Jin-gang, 2013^(81)^ | Descriptive technique | Arch wire bending robot | Proposal to use robots to replace dentists for completing orthodontic wires bending | Manual bending | Bending process analysis and structure design of orthodontic arch wire bending robot | Power function model | S4 |
|  | Zhang, 2013^(87)^ | Descriptive technique | Arch wire bending robot | Propose a third order pure S acc/dec profile with system parameters of arch wire bending robot | Displacement determined by pure S acc/dec profile as the criteria | To establish a third-order pure S acc/dec profile with system parameters of arch wire bending robot | Maximum jerk, maximum acceleration and maximum velocity by software | S4 |
|  | Xia ,  2014 ^(88)^ | Patency  No: CN 103817691 A | Arch wire bending robot | Robot and manipulator for orthodontic appliances |  |  |  | S4 |
|  | Brown, **2015^(^**[**^32^**](#_ENREF_32)**^)^** | Retrospective study | Records of 96 patients treated by different systems | Efficiency of CAD/CAM customized orthodontic appliances | Comparison between direct bonding SLB, indirect bonding SLB, indirect bonding of CAD CAM brackets | To investigate the clinical effectiveness and efficiency of CAD/CAM customized orthodontic appliances compared with direct and indirect bonded stock orthodontic brackets | ABO Cast-Radiograph Evaluation | S4 |
|  | Jin-gang  2015^(93)^ | Descriptive technique | Arch wire bending robot | Structural dynamics in arch wire bending robot | Simulation model of orthodontic arch wire bending | Structural Analysis and dynamics simulation of orthodontic arch wire bending robot | Transient dynamic module of ANSYS/Workbench software | S4 |
|  | Deng, 2015^(86)^ | Descriptive technique | Arch wire bending robot system | Planning and control method  of a robotic system for multi-functional automatic orthodontic arch wire bending | Self-developed simulation platform based on *Robot Operating System* with MoveIt | 1. Propose a motion planning and control method of a robotic system for multi-functional orthodontic arch wire bending 2. Validation in both simulation and physical experiments | 1. MoveIt platform 2. Algorithms supported from Open Motion Planning Library (OMPL) 3. Bending planning package EONsRRT, integrated into OMPL as a user integrated planner | S4 |
|  | Jin-gang, 2016^(89)^ | Descriptive technique | Arch wire bending robot | Design of control system of orthodontic arch wire bending robot | Manual wire bending | Evaluate the proposed design of control system of orthodontic arch wire bending robot in terms of simplicity, speed, automaticity and reliability | LabView software | S4 |
|  | Hartwich, 2016^(99)^ | Case control study | Setup models of 26 consecutive patients | Virtual setups for treatment planning and chairside implementation using custom arch wires fabricated by robots | Models of final outcome | Evaluate precision of implementing virtual setups for orthodontic treatment using CAD/CAM-fabricated custom arch wire | Superimposition methods | S4 |
|  | Xia,  2016^(^[^94^](#_ENREF_94)^)^ | Descriptive technique | Physical orthodontic arch wire bending robot | Development of a robotic system for orthodontic arch wire bending | Developed simulator | To preliminarily validate the developed robot system and its control system and accuracy for orthodontic arch wire bending | The width of orthodontic arch wire D0 can be used to judge the effects of bending control | S4 |
|  | Gilbert, 2016^(^[^95^](#_ENREF_95)^)^ | Descriptive laboratory technique | Super-elastic robot and nitinol tubes | Rapid, reliable shape setting of super-elastic Nitinol for prototyping robots | Traditional furnace-based approaches | 1. Validate and evaluate performance of a more accurate and reliable method of shape setting of super-elastic Nitinol tubes 2. Demonstrate the feasibility of creating general space curves by shape setting a helical tube 3. Study the accuracy of the temperature regulation and the ability of the system to produce the desired final results | 1. K thermocouple of 0.01 in diameter 2. Voltage is measured by set of sense leads 3. Current is measured by 0.5 mΩ high-side shunt resistor and current monitor | S4 |
|  | Jiang,  2017^(90)^ | Descriptive technique | 1. Arch wire bending robot 2. Patient with no clinical experience of orthodontic treatment | A spring- back calculation model for rectangular orthodontic arch wire bending robot | Theoretical model | 1. Analysis of Spring back Mechanism 2. Evaluate the correctness of spring back mechanism model proposed for arch wire bending robot 3. Evaluate maximum and minimum error ratios for arch wire parameters | 1. Instron 5569 electronic universal material testing machine 2. MATLAB software 3. Olympus Bx51m reflected/transmitted light dual- use microscope | S4 |
|  | Breuning,2017 ^(^[^97^](#_ENREF_97)^)^ | Randomized clinical trial | 180 participants (age:12-30 years) | Insignia self-ligating brackets | Standard Damon Q self-ligating brackets | 1. Primary outcome: total treatment duration 2. Secondary outcome: quality of treatment, difference between the planning and the outcome of treatment of the Insignia cases | 1. PAR index 2. Time in minutes | S4 |
|  | Awad, 2018^(^[^28^](#_ENREF_28)^)^ | Retrospective cohort study | Consecutive patients treated with Insignia (n = 21) and Incognito (n = 16) treatment system | Insignia and Incognito customized appliances | Comparison of two treatment systems Insignia and Incognito | To compare the treatment efficacy and efficiency of the two CAD/CAM customized bracket systems Incognito (lingual) and Insignia (labial) | 1- ABO Cast and Radiograph Evaluation system scores  2- Efficiency as assessed by three clinical measures of efficiency  3- Final treatment outcome and the virtual set up | S4 |
|  | Papakostopoulou,2018^(^[^33^](#_ENREF_33)^)^ | Randomized controlled trial | One hundred and eighty patients  requiring fixed orthodontic treatment | Insignia customized orthodontic system | Damon Q noncustomized orthodontic system | 1- The primary outcome: to measure treatment duration.  2- Secondary outcomes were quality of treatment result, the degree of improvement, number of visits from the first visit after bonding to debonding, number of loose brackets, time required for treatment planning and number of complaints. | 1- Peer Assessment Rating (PAR) score;  2- Number of visits from the first visit after bonding to debonding number of loose brackets; time required for treatment planning; and number of complaints. | S4 |
|  | Jin-gang,  2018^(91)^ | Descriptive technique | Arch wire bending robot model | Considering slip warping phenomenon in the robotic bending of arch wires | Theoretical model  of arch wires | 1. Analysis of spring back mechanism 2. Experimentation of orthodontic arch wire   considering slip warping phenomenon   1. Assess accuracy of robotic bending | 1. Spring back measurement device for orthodontic arch wire 2. OLYMPUS metallographic microscope 3. LabVIEW software | S4 |
|  | Jiang,  2018^(92)^ | Descriptive technique | Wire bending robot  with the 3D maxillary information of a patient | 3D digital expression method and robot bending method of orthodontic arch wire | Ideal values based on a set of three-dimensional node maxillary information of a patient | Assess feasibility, accuracy, and error rate of using discrete Bessel curve algorithm to carry out the control point and angle planning | 1- MATLAB simulation  2- LabVIEW software | S4 |
|  | Arino,  2020^(^[^100^](#_ENREF_100)^)^ | Retrospective study | 16 patients encompassing evaluation of 339 teeth. | Virtual prediction of tooth movements that are expressed by the robotically manufactured wires of Sure Smile | Actual outcomes of tip and torque | Evaluate the accuracy of the tip and torque virtual predictions that are expressed by the robotically manufactured wires of Sure Smile | Superimposition of CBCT images using Dolphin software | S4 |
|  | Alzainal,  2020^(^[^34^](#_ENREF_34)^)^ | Single-Center randomized clinical trial | 40 participants | Non-Sliding Lingual Orthodontic Technique (BRIUS) | Preadjusted regular edgewise full fixed appliance | Assessment of alignment and discomfort using non-sliding lingual orthodontic technique (BRIUS) and conventional bracket systems | Regular photographic records | S4 |
|  | Ackerman, 2011^(^[^44^](#_ENREF_44)^)^ | Randomized controlled trial | 9 subjects in the test group (5 males and 4 females) and 10 subjects in the control group  (4 males and 6 females) | Objective measurement of compliance of appliance wear through microsensor monitoring | Control group who was unaware of being monitored | 1. Quantify teenage patient compliance with removable maxillary retention. 2. Compare actual usage vs prescribed usage between subjects who knew they were being monitored via an implanted microsensor in the retainer and those subjects who were unaware of any monitoring. | 1. Patient reporting 2. Microsensor | S5 |
|  | Vanderveken, 2013^(45)^ | Three-months prospective clinical trial | 51 consecutive patients with an established diagnosis of SDB | Objective measurement of compliance in OA therapy by microsensor thermometer | Self-reported OA compliance and self-reported total sleep time (TST) | Assess the safety and feasibility of an objective measurement of compliance during OA therapy using an embedded microsensor thermometer with on-chip integrated readout electronics | 1. Microsensor thermometer 2. Self-reported time by diary polysomnography | S5 |
|  | Kawala, 2013^(^[^46^](#_ENREF_46)^)^ | Clinical trial | 45 children (20 boys and 25 girls) 9.2 years | Microsensors to measure real wear time of removable orthodontic appliances | Subjective measurement of compliance | Objective evaluation of cooperation of patients orthodontically treated with removable appliances | 1. TheraMon microsensor 2. Software | S5 |
|  | Tsomos, 2014^(102)^ | Cross sectional cohort study | 45 white patients | Objective assessment of compliance using microsensor |  | 1. Assess objectively patient compliance with removable orthodontic appliances . 2. Study the effect of possible influential factors as age and sex characteristics, function of the appliance and prescribed appliance wear time on compliance | TheraMon chip  (microsensor) | S5 |
|  | Hyun, 2015^(104)^ | A prospective clinical pilot study | 22 patients | Objective measurement of compliance using SMART microsensor | Control group not informed that they would be monitored | Evaluate the compliance of patients while wearing maxillary Hawley retainers embedded with SMART microsensors | 1. Built-in clock circuit in microsensor 2. Software of microsensor | S5 |
|  | Schäfer, 2015^(105)^ | Clinical cohort study | 141 orthodontic patients  (88 males, 53 females) | Microelectronic wear-time documentation of active removable appliances | Comparison between different age, gender, type of device, location of treatment, and health insurance status | Quantify the wear times of removable appliances during active orthodontic treatment | 1. TheraMon® Sensor 2. TheraMon® Software | S5 |
|  | Gjerde, 2018^(101)^ | Reliability study | Eighty consecutive patients referred to a specialist outpatient sleep medicine clinic | Adherence measured by microsensors | A self- reported diary of MAD use | Test reliability of digitally registered use of a mandibular advancement device (MAD) by a built-in thermal sensor | 1. DentiTrac® sensor 2. Software developed by the manufacturer (Braebon Medical Corporation) 3. Self -reported diary | S5 |
|  | Kirshenblatt  2018^(103)^ |  | 14 volunteers | Thermosensitive microsensors to objectively monitor patient use of  removable oral appliances | 1- TheraMon  2- AIRAID SLEEP  3-DentiTrac microsensors | Evaluate the accuracy of 3 thermo- sensitive microsensors that record “wear time” of removable oral appliances (OAs). | 1. Log sheets of appliance wear 2. Sensors softwares | S5 |
|  | Burgner, 2010^(111)^ | Descriptive technique | 1. Prototype system for robot assisted laser bone ablation 2. A human skull replica 3. A fresh ex vivo pig half‐skull 4. CO_2_ laser | Robot assisted laser bone ablation | Mechanical bone cutting | *Ex vivo* evaluation of accuracy for robot assisted laser bone ablation | 1. Self‐implemented intervention control software 2. Optical tracking system or measurement arm 3. Confocal microscope | S6 |
|  | Vieira, 2010^(112)^ | Phantom and animal cadavar laboratory trials | 1. Light weight robot 2. Phantom patient 3. Swine skull | Stability of robotic holding of the target position while the surgeon drills and fastens the maxilla. | Phantom laboratory simulation | Test light weight robot in laboratory for its stability and usability in orthognathic surgery. | Internal encoders of the robot | S6 |
|  | X. Sun, 2011^(106)^ | Descriptive technique | 1. Image-guided robotic system for automated dental implantation 2. Phantom that is generated from the patient-specific 3D model | Automated dental implantation using image-guided robotics | Other similar systems for dental implantation | Propose the use of a robot for drilling the implant site in preparation for the insertion of the implant | 1. Coordinate Measurement Machine (CMM) 2. Fiducial Registration Error (FRE) 3. Target Registration Error (TRE) values | S6 |
|  | Kasahara Y, 2012^(107)^ | Descriptive technique | Telerobotic assisted bone drilling system | Telerobotic-assisted bone-drilling system using bilateral control with feed operation scaling and cutting force scaling for dental implant surgery | Conventional drilling system | 1. Propose telerobotic-assisted drilling system 2. Evaluate cutting accuracy and reproducibility of the cutting force | 1. Reaction force observer 2. Angular velocity/position error 3. Frequency analysis of the cutting force | S6 |
|  | Syed,  2014^(108)^ | Descriptive technique | Surgical robotic with virtual force feedback | Tele-Robotic assisted dental implant surgery with virtual force feedback | Many CAD/CAM systems that have been developed for preoperative surgical planning | 1. Introduce the integration of the four main areas of surgery to archive safe and secure experimental surgical outcomes 2. Guarantee the flexibility, reproducibility and accuracy of the whole system | 1. Image guided navigation algorithm system 2. Inverse Kinematics algorithm. 3. MIMICS software | S6 |
|  | Sun,  2014^(109)^ | Descriptive technique | Automated image guided dental implantation robot (6- DOF) from Mitsubishi | Automated image-guided surgery for common and complex  dental implants | Manual drilling | 1. Introduce robotic system for automated site preparation for   dental implants   1. Assess accuracy of drilling, volume of bone removed and time needed for removal | 1. Co-ordinate measurement machine (CMM) 2. CBCT 3. Actual volumes of bone removed and milling times 4. Cast molding the shape of the volume removed | S6 |
|  | Gui,  2015 ^(113)^ | Descriptive technique | 1. 7-DOF robotic arm 2. Navigation system 3. Phantom skull model | Navigation and robot-assisted craniofacial surgery | Robotic surgery without navigation | 1. Establish the principal prototype of a novel system for navigation and robot-assisted craniofacial surgery 2. Evaluate its theoretical success | 1. Measure duration of surgery 2. Delay of transmission of instruction from the navigation system to the robotic arm 3. Optical navigation workstation | S6 |
|  | Baek,  2015^(114)^ | Descriptive technique | 1. Er:YAG LASER 2. Surgical robotic arm 3. Navigation system 4. Dummy operating theatre 5. Fully grown female Göttingen minipigs (mean (SD) age 26 (5) months | Robot-guided contact-free laser osteotomy in cranio-maxillo-facial surgery | Piezoelectric (PZE) osteotome and a conventional drill guided by a surgeon | 1. Present first experiences with an integrated, miniaturized laser system guided by a surgical robot 2. Evaluate the performance from the points of view of the workflow, ergonomics, ease of use, and safety features | 1. Software package developed in house uses 2. Photographs and videos | S6 |
|  | Nadjmi. 2016^(118)^ | Cadaver experiment and clinical trial | 1. da Vinci Surgical Robot 2. Cadaver 3. 10 consecutive patients with palatal clefts | Transoral Robotic Cleft Palate Surgery | A group of 30 control patients were subjected to surgery with manual instruments | To assess the safety and feasibility of transoral robotic surgery for the reconstruction of soft palatal clefts | Video and still photography | S6 |
|  | Khan, 2016^(119)^ | Feasibility descriptive technique study | 1. daVinci Surgical System® 2. Adult human cadaver | Trans-oral robotic cleft surgery (TORCS) for palate and posterior pharyngeal wall reconstruction |  | To investigate the technical feasibility of trans-oral robotic cleft surgery (TORCS) to access the posterior pharyngeal wall and palate for potential use in the cleft population. | 1. Still and video photography 2. Subjective assessment | S6 |
|  | Sang-Yoon Woo, 2017^(115)^ | Descriptive technique | 1. Surgical robot 2. Saw bone skull phantom 3. 16 orthognathic surgery patients were applied to the phantom | A new method for enabling a robot to help a surgeon reposition a bone segment and transfer the preoperative plan into the intraoperative phase | Manually reposition the bone from its original position to the target position according to the quantitative deviations provided by the image-guided system | 1. Develop a new method for enabling a robot to assist a surgeon in repositioning a bone segment accurately   Transfer a preoperative virtual plan into the intraoperative phase in orthognathic surgery | 1. CT optical tracking system (OTS) 2. Robot coordinate system | S6 |
| **Sr. No** | Author /Year | **Study Design** | **Participant** | Intervention | **Comparison** | **Outcome Primary/ Secondary** | **Method of measurement** | **Domain** |
|  | Ma,  2019 ^(116)^ | Descriptive technique | 1. Surgical robot 2. Five 3D‐printed mandible models on a volunteer's mandible CT data | Autonomous surgical system comprised of  markerless navigation module and a compact OMS robot and position correlation module | Comparison with other surgical systems like da Vinci and Robodoc | Development and preliminary evaluation of an autonomous surgical system for oral and maxillofacial surgery in terms of accuracy, safety and performance improvement | 1. Robot camera 2. BLENDER software 3. System software | S6 |
|  | Jinyang Wu, 2020^(117)^ | Descriptive technique | 1. 3 skull models 2. Craniomaxillofacial surgical robot system | Robot-Assisted orthognathic surgery | Control group, under the guidance of navigation system, surgeon performed the osteotomies manually on 3 skull models | 1. Introduce the robot-assisted orthognathic surgery 2. Demonstrate the accuracy and feasibility of robot-assisted osteotomy in transferring the preoperative virtual surgical planning (VSP) into the intraoperative phase | Software | S6 |
|  | Cao Z, 2020^(110)^ | Descriptive technique | 1. Surgical robot 2. Four phantoms | Surgical robot system for zygomatic implant placement | One phantom manual operation | 1. Propose the design of a novel surgical robot system for the zygomatic implant placement 2. Evaluate accuracy of the robotic operation | 1. Coordinate transformation algorithm 2. In-house software of CAPPOIS 3. Optical tracking device 4. CT scan alignment between pre and postoperative | S6 |
|  | Hilliard,  2011^(^[^120^](#_ENREF_120)^)^ | PatencyUS20080141534A1 | Robotic system for forming features in orthodontic aligners | A robotic system for forming features in orthodontic aligners includes a control system, a platen for three-dimensional positioning of the aligner, a heating station for selectively heating a small region of the aligner, and a thermoforming station for manipulating the heated region to form a desired feature in the aligner |  |  |  | S7 |
|  | Ishii,  2010^(^[^121^](#_ENREF_121)^)^ | Descriptive technique | 1. Three adult male subjects 2. Massaging robot | Path generator control system and virtual compliance calculator for maxillofacial massage robots | Male subjects without virtual compliance control | Assess feasibility, usefulness, safety and effectiveness of implementation of virtual compliance control in massage robot | 1. Virtual compliance calculator 2. Inverse kinematics calculator. 3. Force sensors | S8 |
|  | Hiraiwa, 2013.^(^[^122^](#_ENREF_122)^)^ | Clinical controlled trial | 1. Sixteen patients with myofascial pain (41 +-15.7 years) and 24 healthy volunteers were enrolled   39.8±12.5 years   1. Massage robot | Efficacy of massage treatment technique in masseter  muscle hardness by robotic experimental approach | Healthy volunteers | 1. Clarify the masseter muscle hardness in patients with myofascial pain 2. Examine their changes after massage (efficacy of massage treatment) 3. Analyze whether the hardness can be an index for mas-sage treatment | Portable muscle  hardness meter | S8 |
|  | Wang X, 2014.^(125)^ | Descriptive technique | Jaw Exoskeleton Rehabilitative model | Development of jaw exoskeleton for rehabilitation of Temporomandibular Disorders | Motion pattern from in-vivo recording of jaw movement from healthy subject | 1. Propose the mechanism, design and construction of a jaw exoskeleton 2. Propose the primary motion of the jaw as a planar movement, forming the design specification in the aspect of the kinematics 3. Reflect the adjustability, the applicability and the safety of the proposed exoskeleton | 1. Articulograph AG500 2. A GUI program is coded in MATLAB software | S8 |
|  | Ariji Y, 2015.^(123)^ | Clinical trial | 1. 41 (TMD) patients, median age: 46 years) 2. Rehabilitation Robot | Masseter and temporal muscle massage treatment using an oral rehabilitation robot | Normal massage | 1. Assess the safety, suitable treatment regimen, and efficacy of masseter and temporal muscle massage treatment using an oral rehabilitation robot. 2. Changes in masseter muscle thickness | 1. Sonograms 2. Frequency of adverse events during the massage 3. Degree of TMJ dysfunction change with massage treatment | S8 |
|  | Sun, 2015^(127)^ | Descriptive technique on cadaver head | 1. Soft Oral Interventional Rehabilitation Robot 2. Cadaver head | A new solution to recover mandibular mobility: a soft oral rehabilitation robot (SORR), which is actuated by a novel type of soft pneumatic actuator (SPA) | Conventional actuators | 1. Predict soft oral rehabilitation robot behavior and eligibility for the application 2. Measure elongation and force output of the SPA in terms of applied pressure and extension speed | 1. Camera 2. Ruler and pressure monitor 3. Videos are processed using motion analysis software (Tracker) 4. Instron measuring setup (Instron®) | S8 |
|  | Rubio, 2015.^(^[^126^](#_ENREF_126)^)^ | Descriptive technique | Rehabilitative robotic exoskeleton model | Redesign of a robotic exoskeleton for rehabilitation of TMD | Preexisting exoskeleton  which uses a helmet fitted onto a patient’s head, as well as a four-bar- linkage which emulates jaw motion during chewing | 1. Assess improvements on features such as general aesthetics, patient comfortability, ease of use as well as safety. 2. Assess the actuation design and motion control 3. Measure force exerted | 1. EMG sensor 2. Force sensors | S8 |
|  | Ariji, 2016^(124)^ | Clinical trial | 1. Thirty-seven TMD patients with myofascial pain, mean age 45 years 2. Massage robot | Sonographic features as efficacy  predictors of robotic massage treatment for masseter and temporal muscle in patients with TMD with myofascial pain | Therapy ineffective group | Detect sonographic predictors for the efficacy of massage treatment of masseter and temporal muscle in temporomandibular disorders (TMDs) patients with myofascial pain | 1. Maximum mouth opening 2. Visual analog scale scores of muscle pain and daily life impediments 3. Sonographic or sonoelastographic images | S8 |
|  | Evans, 2016^(^[^128^](#_ENREF_128)^)^ | Descriptive technique | Neurological rehabilitative robotic model | A Shoulder-mounted robotic exoskeleton for neurological rehabilitation of TMD via Assisted motion of the jaw | Other robotic and exoskeleton rehabilitation robots in literature | Evaluate simplicity, ease of set up, safety, weight, portability, effectiveness of a shoulder-mounted robotic exoskeleton for neurological rehabilitation of TMD | 1. EMG 2. Standalone software | S8 |
|  | Surya S, 2017^(^[^129^](#_ENREF_129)^)^ | Descriptive technique | Rehabilitative robotic model | Robotic exoskeleton for Rehabilitation of TMD via assisted Motion of jaw | Other rehabilitative robots | 1. Record electrical activity of brain 2. Measure muscle activation 3. Identify the force exerted by the patient to move the jaw | 1. Electroencephalography (EEG)sensor 2. Electromyography (EMG) sensor 3. Strain gauge | S8 |
|  | Kalani H, 2018  ^(^[^130^](#_ENREF_130)^)^ | Descriptive technique | Masticatory rehabilitation robot  (Two Gough–Stewart robots) | Dynamic modeling and CPG-based trajectory generation for a masticatory rehab robot | Real masticatory patterns: frequency and amplitude of mastication | 1. Provide a methodology to enable physiotherapists to perform the human jaw rehabilitation for real-time trajectory generation 2. Track and record chewing trajectories 3. Online assessment of chewing pattern, amplitude and velocity | 1. Simi Reality Motion System 2. Simi Motion software 3. Three synchronized cameras 4. Inverse kinematics 5. Fourier analysis   proportional-derivative computed torque controller   1. Gibbs–Appell method 2. MATLAB/Simulink 2012b | S8 |
|  | Kalani, 2019^(^[^131^](#_ENREF_131)^)^ | Descriptive technique | 1. Twenty men  (age: 21±2 years) 2. Chewing robot | Bio-inspired rehabilitation aid: sEMG-CPG approach for online generation of jaw trajectories for a chewing robot | Final robot manipulation experiment | Assess feasibility for a rehabilitation robot with sEMG-CPG approach for online generation of jaw trajectories for a chewing robot | Surface electromyography signals of the masticatory muscles of a tele-operator through sensors | S8 |

Supplement 2. **Studies excluded from the scoping review (n = 46).**

| **Author/Year** | **Reason for exclusion** | **Domain** |
| --- | --- | --- |
| 1. Bi XQ, 2018^(142)^ | Chinese language | 3,4,6 |
| 1. Dallan I, 2019^(143)^ | Less than 5 participants | 6 |
| 1. van der Meer WJ, 2016^(144)^ | A proof of concept | 4 |
| 1. Peacock ZS, 2014^(145)^ | Research summit | 6 |
| 1. Janakiraman N, 2015^(146)^ | Case report | 6 |
| 1. Gracco A, 2013^(147)^ | Case report | 4 |
| 1. Velo S, 2015^(148)^ | Korean language | 4 |
| 1. Grischke J, 2020^(^[^7^](#_ENREF_7)^)^ | Systematic review | 1,2,3,4,6 |
| 1. Iwasaki LR, 2015^(149)^ | Workshop | 4 |
| 1. Das PK, 2019^(150)^ | Review article | 3,5,6 |
| 1. Dimri D, 2020^(151)^ | Review article | 3,4,5,6 |
| 1. Joo S-H, 2014^(152)^ | Korean language | 4 |
| 1. Jiang J , 2016^(153)^ | Review article | 4 |
| 1. Bhat BD, 2017^(154)^ | Review article | 3,5,6 |
| 1. Balan B, 2014^(^[^43^](#_ENREF_43)^)^ | Review article | 5 |
| 1. Jiang J, 2019^(155)^ | Chinese language | 4 |
| 1. Ankita Y, 2019^(156)^ | Review article | 5 |
| 1. Jiang J-g, 2015^(132)^ | Review article | 4,6 |
| 1. Abutayyem H, 2019^(157)^ | Review article | 4 |
| 1. Goraya KS, 2017^(158)^ | Review article | 4 |
| 1. Saifee A, 2019^(159)^ | Review article | 5 |
| 1. Jiang J, 2016 ^(160)^ | Review article | 8 |
| 1. Shin S, 2011^(161)^ | Korean language | 1 |
| 1. Min H-H, 2018^(162)^ | Korean language | 3 |
| 1. Khatria H, 2019 ^(^[^35^](#_ENREF_35)^)^ | Review article | 5 |
| 1. Wu Q , 2019^(163)^ | Review article | 4,6,8 |
| 1. Ahmad S, 2016^(133)^ | Review article | 4 |
| 1. Raj S, 2020^(164)^ | Review article | 1,3,4,6,7 |
| 1. Kumar P, 2017^(^[^47^](#_ENREF_47)^)^ | Review article | 3,4,6 |
| 1. Kerautret Y, 2020^(165)^ | Review article | 8 |
| 1. Tsuda H, 2017^(166)^ | Review article | 5 |
| 1. Kirshenblatt S, 2018^(167)^ | Review article | 5 |
| 1. Gracco A, 2011^(^[^30^](#_ENREF_30)^)^ | Descriptive paper | 4 |
| 1. Gilbert A, 2011 ^(^[^29^](#_ENREF_29)^)^ | Descriptive paper | 4 |
| 1. Kawana H, 2015 ^(168)^ | Presentation | 6 |
| 1. Nambi N, 2016 ^(169)^ | Review article | 5 |
| 1. Zhang Y, 2012^(170)^ | Chinese language | 4 |
| 1. Gambhir RS, 2013^(^[^37^](#_ENREF_37)^)^ | Review article | 5 |
| 1. Govindankutty D, 2015^(^[^38^](#_ENREF_38)^)^ | Review article | 5 |
| 1. Bansal A, 2016^(^[^48^](#_ENREF_48)^)^ | Review article | 6 |
| 1. Jiang JX, 2013^(171)^ | Unable to retrieve full article | 4 |
| 1. Jiang JG, 2015^(172)^ | Unable to retrieve full article | 4 |
| 1. YC G, 2015^(173)^ | Unable to retrieve full article | 4 |
| 1. Schott TC, 2011 ^(174)^ | Unable to retrieve full article | 5 |
| 1. Shahroom NSB, 2020^(175)^ | Review article | 4 |
| 1. Lee W, 2015^(176)^ | Descriptive paper | 4 |
